# European Biophysics Journal - Special Issue Celebrating the 70th Anniversary of the Institute of Biophysics, Czech Academy of Sciences

**DOI:** 10.1007/s00249-025-01804-5

**Published:** 2025-11-17

**Authors:** Aleš Kovařík, Martin Hof, Eva Bártová

**Affiliations:** 1 Department of Molecular Epigenetics, Institute of Biophysics, Academy of Sciences of the Czech Republic, Královopolská 135, Brno, 612 00 Czech Republic; 2 Department of Biophysical Chemistry, J. Heyrovsky Institute of Physical Chemistry, Academy of Sciences of the Czech Republic, 2155, 182 00 Dolejškova, Libeň, Praha 8, Czech Republic; 3 Department of Cell Biology and Epigenetics, Institute of Biophysics, Academy of Sciences of the Czech Republic, Královopolská 135, Brno, 612 00 Czech Republic

This special issue of the *European Biophysics Journal* is dedicated to the 70th anniversary of the Institute of Biophysics of the Czechoslovak (now Czech) Academy of Sciences (IBP), founded on January 1, 1955. The issue comprises four original and four review articles focused on biophysics or the results obtained using biophysical methods, including CD spectroscopy, computational chemistry, fluorescence recovery after photobleaching (FRAP), or other fluorescent-protein technologies. The special issue of the *European Biophysics Journal*, dedicated to the anniversary of the establishment of biophysics in the Czech Republic, highlights the significant progress achieved in biophysical research in the country, as well as the foundation of the Czech Biophysical Society (https://www.cbpa.cz/en.html).

The opening review article by **Eva Bártová and colleagues** provides a comprehensive overview of IBP’s history, tracing its establishment and evolution across seven decades. The authors explore the Institute’s development from scientific, personal, and socio-political perspectives, highlighting its major research domains, from early studies in radiobiology (effects of ionizing radiation), through molecular biophysics (nucleic acids and chromatin), and cell biology (nucleus and epigenetics), to current approaches employing super-resolution microscopy and computational biophysics. The review underscores the Institute’s central role in advancing biophysics within the Czech Republic and its contributions to the global scientific community through methodological innovations and applications in biology and medicine. It also reflects on how the field of biophysics has matured in the Czech Republic from its origins in radiobiological physics to a fully interdisciplinary discipline bridging physics, chemistry, biology, and computation.

Below, we briefly summarize the individual contributions included in this special issue:

**Miroslav Fojta and Jan Paleček** commemorate the remarkable 63-year scientific career of **Emil Paleček**, who joined IBP in 1955 and remained active there until his passing in 2018. Their article presents a hybrid format combining a biographical sketch with a retrospective of Paleček’s major scientific achievements in electrochemistry, nucleic acids, proteins, and glycans. The authors situate his discoveries within the evolving context of bioelectrochemistry, illustrating how his pioneering work shaped and was influenced by developments in the field both in Czechia and internationally. 

The review by **Jiří Toufar and colleagues** traces the trajectory of radiobiology research at IBP, with particular focus on studies involving irradiated mice and their progression toward modern molecular and biophysical approaches. Key themes include: (i) the transition from in vivo radiobiological experiments to molecular-level investigations of DNA damage, repair, and chromatin effects; (ii) the interplay between radiation and chromatin architecture, including higher-order nuclear organization; (iii) comparative effects of photon versus high-LET (linear energy transfer) particle radiation on DNA damage responses; and (iv) the transformative impact of technological advances in microscopy, molecular assays, and computational modeling in contemporary radiobiology. 

**Petra Školáková and her team** focus on G/C-rich DNA sequences that can form alternative secondary structures beyond the canonical B-DNA double helix, namely, guanine quadruplexes (G4) and cytosine i-motifs (iM). Using circular dichroism and absorption spectroscopy, as well as melting experiments, they examined the equilibrium and transitions between these conformations under varying sequence contexts and conditions (pH, loop length). Their findings reveal that the double helix often remains dominant, yet specific sequence features and environmental factors can shift the equilibrium toward G4 or iM formation.

**Anne Cucchiarini and colleagues** investigate how different cations, in type and concentration, influence the stability of DNA G-quadruplexes compared with canonical duplex DNA. A bioinformatic analysis revealed an enrichment of potential G4-forming sequences in genomes of halophilic (salt-tolerant) organisms. Complementary experimental studies (FRET and UV melting) on multiple G4 and hairpin duplex sequences under varied ionic conditions demonstrate that increasing ionic strength enhances the stability of both duplex and G4 structures, though the stabilizing effect is more pronounced for G4s. 

The study by **Paolo Fagherazzi and colleagues** examines how specific TP53 (p53) missense mutations affect recruitment of the non-homologous end-joining (NHEJ) factor TP53BP1 (53BP1) to DNA double-strand breaks. Analyzing three common hotspot mutations (R248W, R273C, and G245S), the authors found that the R248W and R273C variants impair the recruitment of 53BP1 and its partner RIF1 compared with wild-type p53, while the G245S mutation exhibits a distinct pattern, partially preserving 53BP1 localization but disrupting downstream signaling events such as RIF1 and BRCA1 foci formation.

**Jana Sochorová and colleagues** explore the behavior of ribosomal RNA genes (rDNA) in human breast cancer cells exposed to γ-irradiation and under senescent conditions. Using advanced genomic, transcriptomic, and confocal microscopy approaches, they show that irradiation-induced senescence is characterized by nucleolar fragmentation and accumulation of polyadenylated rRNA species from noncoding rDNA regions. Despite these changes, rDNA sequence and transcriptional variants remain stable, indicating that altered rRNA processing, rather than gene sequence variation, drives nucleolar disruption. These insights may inform clinical applications of radiotherapy in oncology. 

In their opinion article, **Eduard Kejnovský and colleagues** advocate for a re-evaluation of evolutionary complexity, emphasizing the crucial role of non-cellular genetic entities, including viruses, plasmids, and transposons, and their role in driving evolutionary innovation. They propose the term *Acytota* to designate a kingdom of life encompassing these non-cellular replicators and argue that their dynamic interactions with cellular life have profoundly shaped genome evolution, epigenetic regulation, and the arms race between parasitic elements and their hosts. Finally, we extend our sincere gratitude to **Professor Robert Gilbert**, Editor-in-Chief of the *European Biophysics Journal*, for his invaluable support, enthusiasm, and guidance throughout the preparation of this special issue.

## Data Availability

No datasets were generated or analysed during the current study.

